# New opportunities for nanoparticles in cancer immunotherapy

**DOI:** 10.1186/s40824-018-0133-y

**Published:** 2018-09-26

**Authors:** Wooram Park, Young-Jae Heo, Dong Keun Han

**Affiliations:** Department of Biomedical Science, CHA University, 335 Pangyo-ro, Bundang-gu, Seongnam-si, Gyeonggi 13488 Republic of Korea

**Keywords:** Cancer immunotherapy, Nanoparticle, Cancer antigens, Tumor microenvironment (TME), Biomaterials

## Abstract

**Background:**

Recently, cancer immunotherapy has become standard for cancer treatment. Immunotherapy not only treats primary tumors, but also prevents metastasis and recurrence, representing a major advantage over conventional cancer treatments. However, existing cancer immunotherapies have limited clinical benefits because cancer antigens are often not effectively delivered to immune cells. Furthermore, unlike lymphoma, solid tumors evade anti-cancer immunity by forming an immune-suppressive tumor microenvironment (TME). One approach for overcoming these limitations of cancer immunotherapy involves nanoparticles based on biomaterials.

**Main body:**

Here, we review in detail recent trends in the use of nanoparticles in cancer immunotherapy. First, to illustrate the unmet needs for nanoparticles in this field, we describe the mechanisms underlying cancer immunotherapy. We then explain the role of nanoparticles in the delivery of cancer antigens and adjuvants. Next, we discuss how nanoparticles can be helpful within the immune-suppressive TME. Finally, we summarize current and future uses of nanoparticles with image-guided interventional techniques in cancer immunotherapy.

**Conclusion:**

Recently developed approaches for using nanoparticles in cancer immunotherapy have enormous potential for improving cancer treatment. Cancer immunotherapy based on nanoparticles is anticipated not only to overcome the limitations of existing immunotherapy, but also to generate synergistic effects via cooperation between nanoparticles and immune cells.

## Background

Cancer is a global disease with a high mortality rate [1]. Traditional chemotherapy and radiotherapy have limited therapeutic effects against cancer, and these approaches are associated with severe side effects and high risk of recurrence. Recently, immunotherapeutic agents have yielded promising results in clinical cancer treatment, and have therefore attracted close attention from clinicians and cancer patients worldwide [2–4]. The advantage of cancer immunotherapy is that it not only can treat primary cancer, but can also prevent metastasis and recurrence [5]. Consequently, cancer immunotherapy has become a standard treatment option for cancer patients. Various immunotherapeutic antibodies and cell therapeutics are in active development all over the world [6, 7]. In particular, immune checkpoint blockades have been developed using antibodies against cytotoxic T-lymphocyte antigen 4 (CTLA-4), programmed cell death-1 (PD-1), and programmed cell death ligand-1 (PD-L1), and PD-1 inhibitors have been approved for melanoma, non–small cell lung cancer, renal cancer, Hodgkin’s lymphoma, bladder cancer, and head and neck cancer (Table 1). However, existing cancer immunotherapy has its own side effects, including autoimmune disease [3]. Moreover, immunotherapy is less effective against solid tumors than lymphoma [8, 9] because these cancers create an abnormal extracellular matrix (ECM) that is difficult for immune cells to penetrate, as well as forming an immune-suppressive tumor microenvironment (TME) [10–13].

**Table 1:** Major cancer immunotherapeutics approved by the US Food and Drug Administration (FDA)

Drug (Trade Name)	Drug Type (Target)	Targeted Cancer	FDA Approval Date
Ipilimumab (Yervoy®)	Monoclonal antibody (CTLA-4)	Advanced melanoma	March 2011
Pembrolizumab (Keytruda®)	Monoclonal antibody (PD-1)	Advanced refractory melanoma and non–small cell lung cancer	September 2014
Nivolumab (Opdivo®)	Monoclonal antibody (PD-1)	Unresectable or metastatic melanoma squamous non–small cell lung cancer	December 2014
Atezolizumab (Tecentriq®)	Monoclonal antibody (PD-L1)	Locally advanced or metastatic bladder cancer	May 2016
Avelumab (Bavencio®)	Monoclonal antibody (PD-L1)	Locally advanced or metastatic bladder cancer	May 2017
Durvalumab (Imfinzi®)	Monoclonal antibody (PD-L1)	Locally advanced or metastatic bladder cancer	May 2017

*CTLA*-4 cytotoxic T lymphocyte–associated protein-4, *PD*-1 programmed cell death-1, *PD-L*1 programmed cell death ligand-1

Recent efforts have used biomaterial-based nanoparticles to address the limitations of existing cancer immunotherapies [14–17]. Nanoparticles have been extensively studied in the field of drug delivery in light of their ability to efficiently deliver drugs to target sites, protect drugs from endogenous enzymes, and remain in the circulation for long periods of time.

Three factors are important for effective cancer immunotherapy. First, cancer antigens must be effectively transferred to immune cells, especially antigen-presenting cells (APCs). Second, when an adjuvant is delivered to immune cells along with the cancer antigens, it must induce an anti-cancer immune response. Third, the immune-suppressive TME must be modulated to respond to the anti-cancer immunotherapeutics. Nanotechnology can be used for each of these aspects, and therefore has the potential to effectively induce anti-cancer immune responses. Here, we review recent trends in the use of biomaterial-based nanoparticles in cancer immunotherapy.

## Mechanism of cancer immunotherapy

**Fig. 1:**
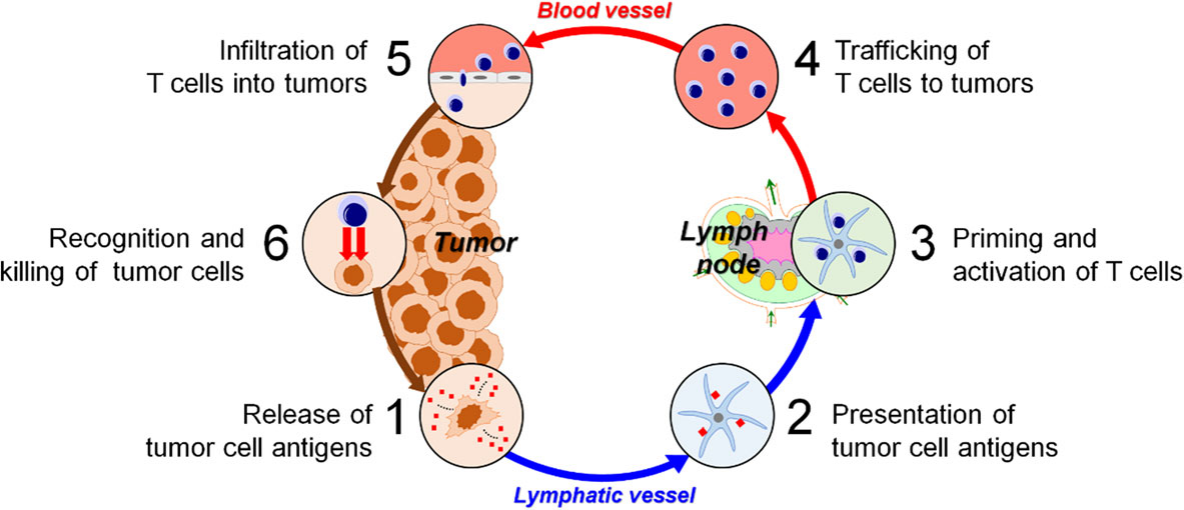
Cancer–immunity cycle. Tumor antigens released from tumor cells are recognized by antigen-presenting cells (APCs). Matured APCs migrate to the lymph nodes, leading to priming and proliferation of T cells. T cells activated by APCs are transferred to tumor tissues, where they kill tumor cells. Finally, tumor antigens from killed cancer cells induce another round of the immune response, leading to a cancer–immunity cycle

Before describing the use of nanoparticles to treat cancer, we must first understand the mechanisms underlying cancer immunotherapy. As shown in Fig. 1, cancer immunotherapy removes tumor cells through a cancer–immunity cycle. When cancer cells die through apoptosis or necrosis, tumor antigens are captured by APCs, such as dendritic cells, and presented on major histocompatibility complex (MHC). Dendritic cells bearing the cancer antigens move to the lymph nodes, where they prime immature T cells. Subsequently, activated tumor-specific cytotoxic T lymphocytes (TCLs) infiltrate the tumor site and recognize tumor cells by interacting with T-cell receptors and MHC complexes. Finally, effector T cells kill cancer cells by inducing apoptosis, and the resultant release of additional cancer antigens strengthens the immune response. These processes all play an important role in inducing effective anti-cancer immunity. However, several obstacles limit the therapeutic efficacy of the anti-cancer immune response.

When pro-inflammatory cells such as M1-polarized macrophages, which are tumoricidal, kill tumor cells, the dead cells release immunosuppressive factors including IL-10, TGF-β, and sphingosine-1-phosphate (S1P), causing macrophages to repolarize from M1 to M2 [18–20]. Furthermore, apoptotic tumor cells secrete chemoattractants, such as monocyte chemoattractant protein-1 (MCP-1) and bombesin (BN), that trigger infiltration of monocytes into the TME [19, 21–23]. These monocytes differentiate into tumor-associated macrophages (TAMs), which promote tumor growth and enable the tumor to avoid immune surveillance [19, 21–23]. Moreover, infiltration of myeloid-derived suppressor cells (MDSCs) following tumor cell death contributes to suppression of the cancer immune response. MDSCs secrete anti-inflammatory cytokines, resulting in activation of immunosuppressive regulatory T (Treg) cells. Tregs impede dendritic cell maturation, which supports tumor remission [19, 24–26]. To make matters worse, when TCLs arrive at tumor tissues to kill the tumor cells, immune-suppressive molecules on tumor cells (e.g., PD-L1 and PD-L2) and T cells (e.g., CTLA-4 and PD-1) suppress the activation of TCLs, enabling the tumor to evade the anti-cancer immune response and ultimately limiting the efficacy of the immunotherapy [2, 19, 27–30].

Together, these phenomena highlight the importance of overcoming the limitations of current cancer immunotherapies. Nanomaterials can intervene in multiple stages of immunotherapy treatments to further strengthen anti-cancer immunity.

## Delivery of cancer antigens through nanoparticles

To induce tumor immunity, tumor antigens must be effectively transferred to APCs. Tumor-associated antigens can be classified as tumor-associated antigens (TAAs) or tumor-specific antigens (TSAs). TAAs are antigens that appear more often in cancer cells than in normal cells, or at the differentiation stage of cancer cells. Because TAAs are also expressed on normal cells, the use of these antigens as immunotherapeutic targets may cause an autoimmune reaction. By contrast, TSAs (also called neo-antigens) are exclusively present in cancer cells. Consequently, approaches that target TSAs are free from immune tolerance and autoimmune problems. However, these native tumor antigens are easily degraded by enzymes in the body, and have low immunogenicity due to their low efficiency of transfer to immune cells. Because the immune response is primarily initiated in secondary lymphoid organs, tumor antigens must be efficiently delivered to the lymph nodes in order to effectively induce an anti-cancer immune response. Accordingly, nanoparticles have been extensively studied as delivery vehicles for safely transferring tumor antigens to lymph nodes [31]. In this context, nanoparticles have two notable advantages: they can protect tumor antigens from degradative enzymes in the body, and also promote selective delivery to the lymph nodes. Upon delivery, nanoparticles encapsulating cancer antigens are effectively internalized into APCs [32]. Although nanoparticle-mediated delivery systems solve many of the problems described above, several considerations should be taken into account when using such systems, particularly in regard to nanoparticle design.

The delivery of nanoparticles for lymph node targets depends sensitively on particle size, surface charge, shape, and hydrophobicity [33–36]. In particular, nanoparticle size is the most important factor governing the transfer of cancer antigens. Small nanoparticles (< 5 nm) may leak out of blood vessels during circulation, whereas large nanoparticles (> 100 nm) can be trapped in the ECM and are restricted to lymph nodes. By contrast, medium-sized nanoparticles (~ 5–100 nm) persist in the circulation and are effectively delivered to the lymph nodes through lymphatic vessels. Swartz et al. showed that 20–45 nm poly(propylene sulfide) (PPS) nanoparticles were absorbed into lymphatic vessels and delivered to lymph nodes up to 120 h after administration [37]. Moreover, approximately half of these particles were present in lymph node dendritic cells and APCs [15, 37]. Moreover, Hubbell et al. reported that, after intradermal injection of 25 and 100 nm nanoparticles, the 25 nm nanoparticles were efficiently delivered to draining lymph nodes through the interstitial fluid and lymphatic system, whereas only 10% of the 100 nm nanoparticles were successfully delivered [15, 35]. Therefore, medium-size (~ 5–100 nm) nanoparticles are optimal for efficient delivery of tumor antigens to the lymph nodes. It is also possible to utilize active transport by modifying nanoparticles with chemical ligands, such as mannose, to more selectively deliver nanoparticles to the lymph nodes.

Along with size, particle shape is another important factor that influences lymph node draining of nanoparticles. Previous nanoparticle formulations were mainly spherical, but recent advances in nanoparticle engineering have generated a wide repertoire of other shapes (rods, prisms, cubes, stars, and discs) [38]. It is generally accepted that non-spherical particles, with higher aspect ratios, have higher blood circulation times, prolonged margination effects [39, 40], and higher penetration capacities within solid tissues and tumors [41].

Besides particle size and shape, the surface charge of the carrier plays a significant role in cellular internalization and activation of the immune response [42]. Surface charge may affect the uptake of nanoparticles by cells. Generally, positively charged nanoparticles generate a higher immune response than those that are neutral or negatively charged. However, positively charged nanoparticles exhibit reduced tissue permeability, probably because they are often immobilized in the negatively charged ECM [43]. When targeted dendritic cells are localized at the site of injection, it is easier for them to take up cationic nanoparticles than neutral or anionic charged particles. Conversely, carriers with a positive surface charge can be problematic for lymphatic transport and trafficking in vessels because they can induce hemolysis and platelet aggregation [44], resulting in premature antigen release and differences in cellular uptake and antigen transfer kinetics [45].

Recent research has revealed that, among biomaterials, polymers that contain hydrophobic domains, such as PLGA and chitosan, have intrinsic adjuvant activity and are capable of activating immune cells even without additional signals [46, 47]. For instance, nanoparticles made of amphiphilic poly(gamma-glutamic acid) (PGA) exhibit increases in antigen uptake and activation of dendritic cells in vitro, as well as cellular responses in vivo, as a function of increased side-chain hydrophobicity [48].

## Delivery of adjuvant through nanoparticles

An adjuvant is a molecule that increases immunogenicity, which is sometimes lacking in tumor antigens when presented alone. Adjuvants resemble molecules produced by infectious pathogens and recognized by pattern recognition receptors (PRRs) [49]. For example, 3-O-desacyl-4′-monophosphoryl lipid A (MPLA), lipopolysaccharide (LPS), CpG oligodeoxynucleotides (ODNs), polyinosinic:polycytidylic acid (poly I:C), and agonists of the stimulator of IFN genes (STING) are adjuvants commonly used in cancer immunotherapy. When they are internalized into APCs with tumor antigens, adjuvants promote the anti-cancer immune response. Hence, various strategies have been developed to effectively deliver adjuvant into APCs using nanoparticles [50]. Because CpG ODNs, poly I:C, and STING agonists are negatively charged, they form electrostatic complexes with positively charged nanoparticles or polymers, and then transfer them into APCs via endocytosis, thereby promoting anti-cancer immunity [51, 52]. The use of nanoparticles to deliver adjuvants, along with antigen, to the cytoplasm of APCs plays a crucial role in inducing strong antigen-specific T-cell responses [53]. Furthermore, when cancer antigen delivery based on nanoparticles is combined with an immune checkpoint blockade, the anti-cancer immune response can be further improved. Thus, nanoparticle strategies have enormous potential for treatment of multiple types of blood and solid cancer.

**Fig. 2:**
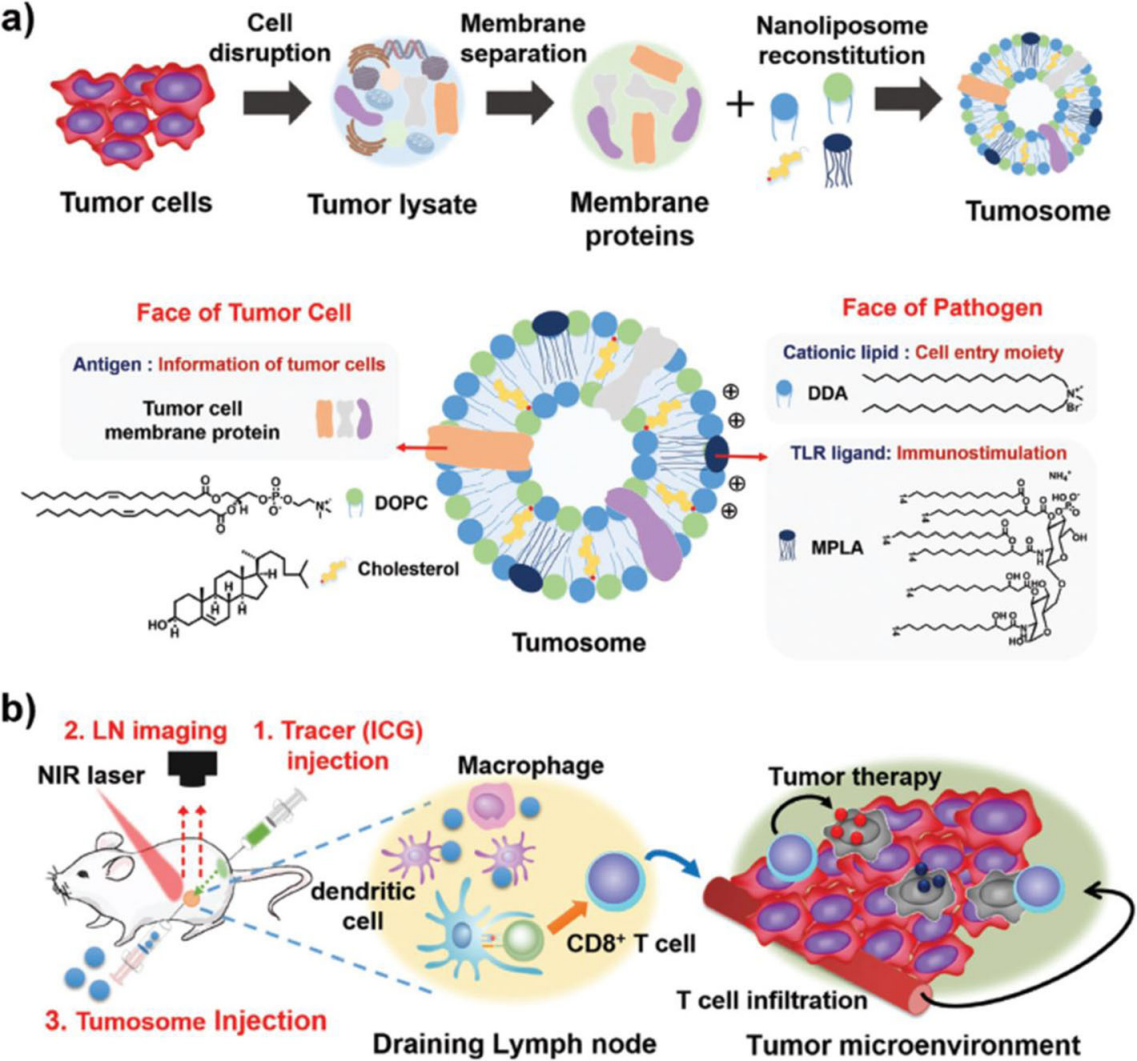
Schematic illustration of multifaceted immunomodulatory nanoliposome (tumosome)-based cancer immunotherapy. **a** Multifaceted tumosomes consist of tumor cell membrane proteins, as tumor-associated antigens; two immunostimulatory adjuvants (3-O-desacyl-4′-monophosphoryl lipid A [MPLA] and dimethyldioctadecylammonium bromide [DDA]), as pathogen characters; and helper lipids (1,2-dioleoyl-sn-glycero-3-phosphocholine [DOPC] and cholesterol). **b** Image-guided cancer immunotherapy. First, to determine the exact position of tumor-draining lymph nodes, near infrared (NIR) tracer (indocyanine green, ICG) is injected, Second, tumor-draining lymph nodes are identified using NIR optical imaging. Third, tumosomes are injected into the lymph node with the guidance of NIR imaging (reprinted with permission from Ref [54]; © 2017 Wiley-VCH)

Recently, the Lim group developed multifaceted immunomodulatory nanoliposomes that can simultaneously deliver cancer antigens and adjuvants (Fig. 2) [54]. These 100 nm nanoparticles, denoted “tumosomes,” contain cancerous membrane proteins (cancer antigens) and two immunostimulatory adjuvants: MPLA as a danger signal, and dimethyldioctadecylammonium (DDA) as a cell-invasion moiety. Tumosomes can effectively increase antigen-specific anti-tumor immunity. Injection of tumosomes into mouse tumor models inhibits tumor growth and significantly improves survival. Although this approach could cause autoimmunity against self-antigens, this could be overcome by addition of TSAs. Also, this strategy could be used together with other therapeutic modalities, such as chemotherapy, gene therapy, and cell-based therapy (e.g., T cells, natural killer cells, etc.), to further increase its therapeutic efficacy.

## Modulation of the immune-suppressive TME using nanoparticles

**Fig. 3:**
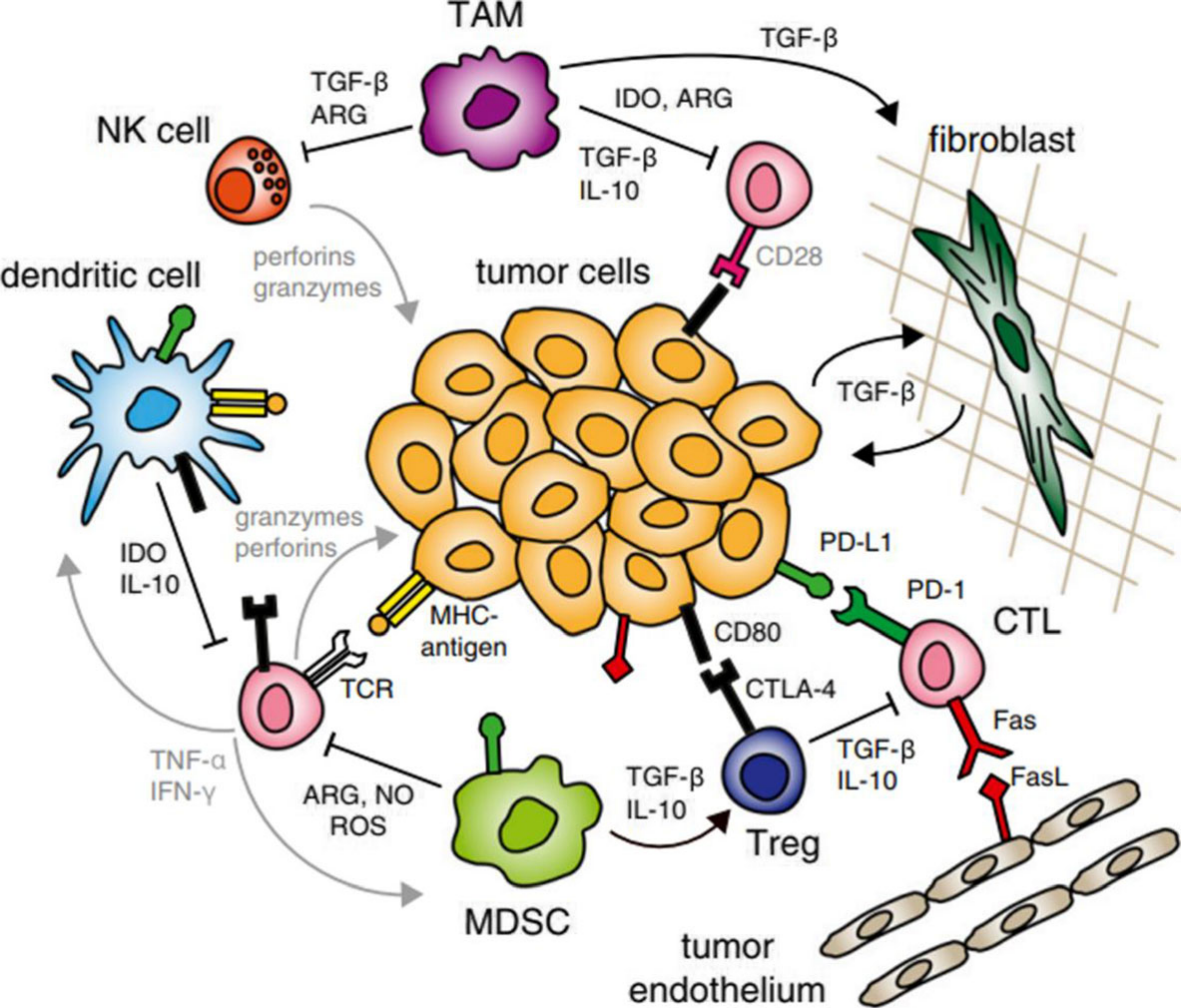
Immune-suppressive tumor microenvironment (TME). Cancer immunity mediated by CTLs is suppressed by compounds secreted by immune cells recruited to the tumor. Cancer cells also express surface molecules that contribute to anergy and exhaustion of anti-cancer immune cells (reprinted with permission from Ref [55]; © 2015 National Academy of Sciences)

Tumors can promote the growth and metastasis of cancer cells by creating an immunosuppressive TME. Consequently, modulation of this environment is an important strategy in cancer immunotherapy (Fig. 3) [55].

Tregs are immunosuppressive T cells that can inhibit the activity of anti-tumor T-effector cells. Tregs play a role in preventing autoimmune disease through immune tolerance to autoantigens, but in the context of cancer, they can inhibit immune cells in the TME and weaken anti-cancer immune activities. To induce anti-tumor immunity, it is possible to functionally suppress or even eliminate the Tregs [56]. For example, a common approach in cancer immunotherapy is controlling Treg function through a checkpoint blockade (e.g., anti–CTLA-4). In addition, nanoparticles capable of targeting Tregs can be engineered to remove Tregs from the TME [57].

TAMs are immune cells that are present in large numbers in the TME. These cells produce high levels of IL-10 and TGF-β immunoregulatory cytokines, and inhibit anti-cancer immune responses by producing inflammatory cytokines such as IL-12, IL-1b, TNF-α, and IL-6. Accordingly, elimination of TAMs in the TME is important for effective cancer immunotherapy. Recently, several attempts have been made to enhance the effectiveness of anti-cancer therapy using surface-modified nanoparticles capable of targeting and killing TAMs.

TGF-β, a cytokine that is overexpressed in breast, liver, and lung cancer, inhibits immune cell activation, maturation, and differentiation. Thus, inhibition of the TGF-β signaling pathway in the TME may induce an immune response to the tumor. Recently, TGF-β inhibitors encapsulated in lipid nanoparticles were delivered to the TME, inducing both innate and adaptive immune responses, resulting in suppression of tumor growth and improved survival in mice with metastatic melanoma.

MDSCs are tumor-suppressor cells frequently found in the TME of breast, lung, gastrointestinal, and liver cancers. MDSCs release IL-10, ARG1, NOS2, and indoleamine 2,3-dioxygenase (IDO) to activate Tregs and suppress other immune cells. Therefore, elimination of MDSCs could dramatically improve cancer immunotherapy. Nanoparticles can deliver immunomodulators to the TME, either through passive transport via the enhanced permeability and retention (EPR) effect or active transport via various targeting ligands. Therefore, delivery of immune-modulating drugs to the TME could improve the overall anti-cancer immune response with minimal systemic side effects.

**Fig. 4:**
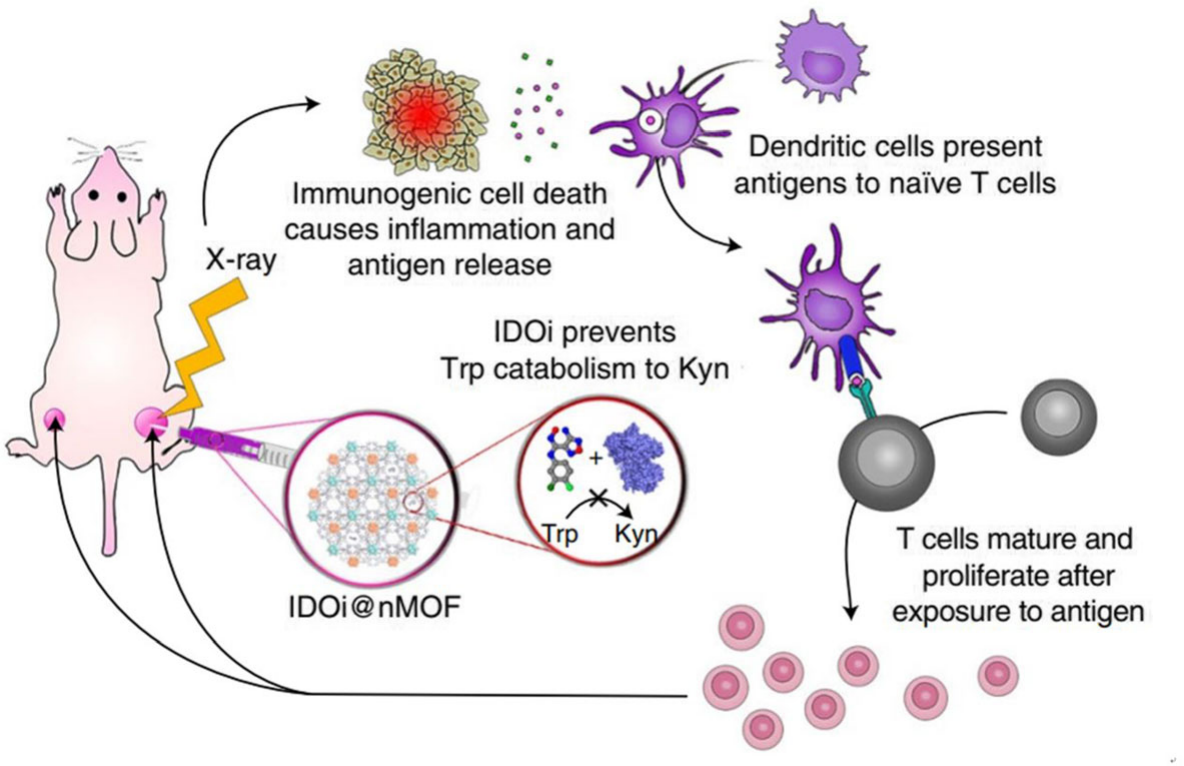
Nanoscale metal–organic framework (nMOFs) enable synergistic radiotherapy–radiodynamic therapy (RT–RDT) and immunotherapy using extremely low doses of X-rays. Indoleamine 2,3-dioxygenase inhibitor nMOF (IDOi@nMOF) was intratumorally injected into the right-side tumors of mice bearing bilateral subcutaneous tumors. Upon low-dose X-ray irradiation, IDOi@nMOF eradicated the right (irradiated) tumors in two ways: via synergistic RT–RDT, which caused both apoptosis and necrosis of cancer cells, and via immunotherapy by IDOi released from the nMOF, which overcame the suppressive tumor microenvironment by preventing catabolism of tryptophan (Trp) to kynurenine (Kyn) and subsequent T-cell anergy. Importantly, systemic IDOi activity combined with local RT–RDT induced immunogenic cell death, and antigen release led to the effective expansion and tumor infiltration of functional CD8^+^ T cells, which effectively suppressed or eradicated the left-side (untreated) tumors (reprinted with permission from Ref [58]; © 2018 Nature Publishing Group)

Recently, the Lin group reported a novel strategy for overcoming the immune-suppressive TME by combining nanotechnology and various therapeutic modalities (Fig. 4) [58]. Their study combined nanoscale metal–organic framework (nMOF)-enabled radiotherapy–radiodynamic therapy, an IDO inhibitor, and checkpoint blockade immunotherapy to control the TME and improve the efficacy of cancer immunotherapy [59]. In a mouse tumor model, injection of nMOFs into the tumor followed by low-dose X-ray irradiation resulted in elimination of the local tumor. Simultaneous treatment with nMOF and IDO inhibitor induces abscopal responses [60–62], enabling successful treatment of distal tumors. This approach represents a new strategy for overcoming the limitations of existing cancer immunotherapy.

## Image-guided local cancer immunotherapy

To date, hundreds of different nanoparticles have been developed to treat cancer, but their clinical applications remain limited. According to a 2016 report, nanoparticles developed over the past decade deliver only 0.7% [median] of the dose injected through the intravenous (IV) route, specifically in the context of solid tumors [63]. The limited efficacy of tumor targeting is related to the TME, which was described above. Abnormal tumor blood vessels, tumor heterogeneity, reticuloendothelial system (RES) sequestration, and tumor-intrinsic barriers greatly diminish the effect of tumor treatment by limiting the entry of nanoparticles into the tumor site. Accordingly, novel strategies in which nanoparticles are delivered to a tumor site through local rather than systemic administration have attracted a great deal of interest among both biomedical engineers and clinicians [64–66].

In interventional oncology, a field derived from interventional radiology, image guidance is used to diagnose and treat cancer in a minimally invasive fashion [67]. Local delivery of therapeutics to tumors located in various organs can be achieved through the use of medical imaging devices such as X-rays, ultrasound, computed tomography (CT), fluorescent optical imaging, or magnetic resonance imaging (MRI) in conjunction with minimally invasive intravascular catheters or biopsy needles. The benefits of image-guided local therapy include a dramatic reduction in drug dose, which in turn ameliorates side effects, promotes rapid recovery, and decreases cost [68].

Due to their inherent physicochemical properties, nanoparticles have been used in the development of multiple imaging agents. For example, iron oxide nanoparticles have been used in a variety of biomedical applications because they are synthesized from in vivo rich metals (i.e., iron) and can be used as MRI contrast agents [69–72]. Gold nanoparticles have high X-ray absorption and can be used as CT contrast agents [73, 74]. Therefore, it should be possible to develop local injectable therapeutics suitable for use in medical imaging by adding such functional nanoparticles.

**Fig. 5:**
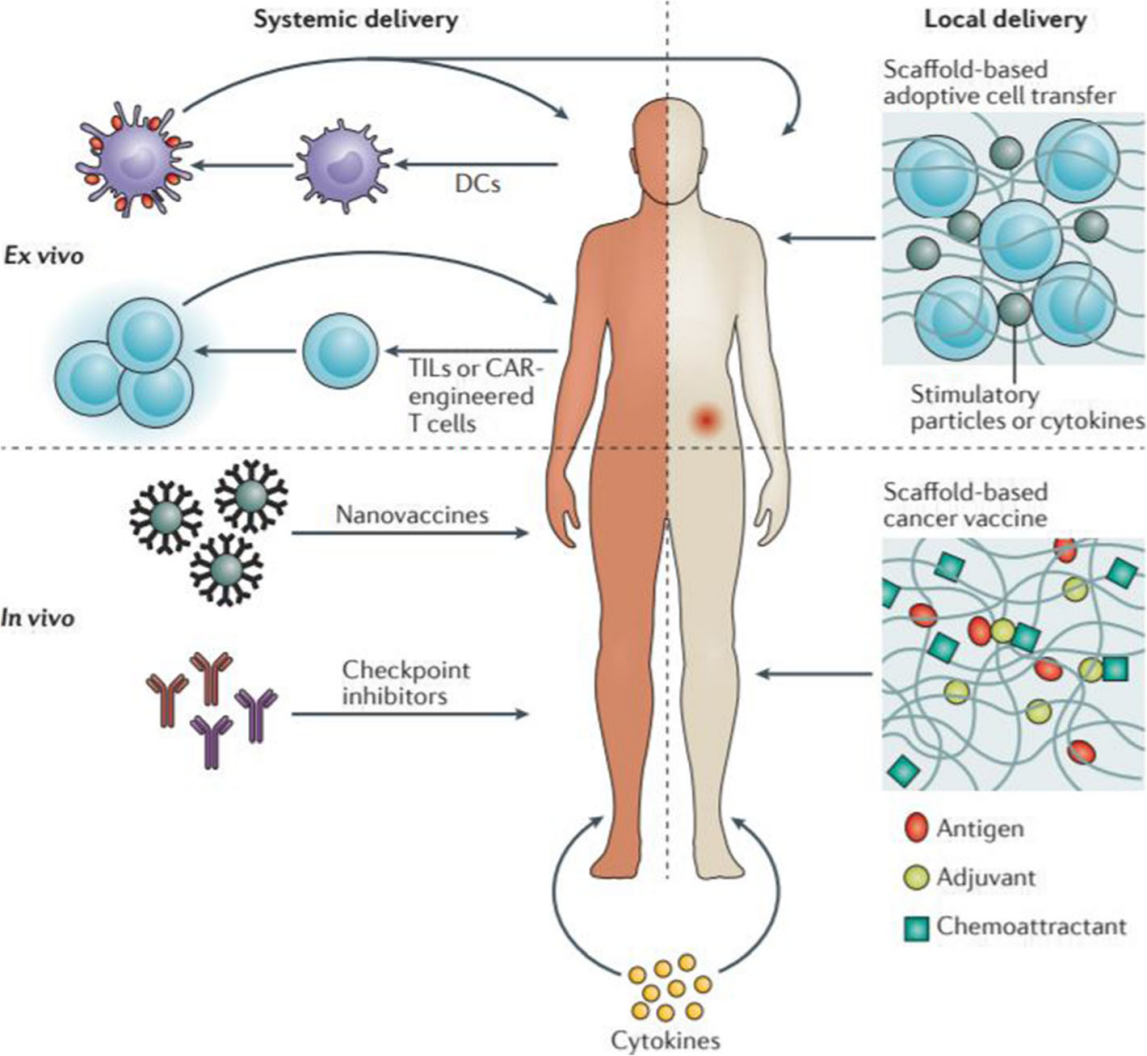
Synthetic immune niches act locally to control the anti-cancer immune response. Current immunotherapeutic strategies are often delivered intravenously, resulting in systemic exposure to immunostimulatory agents and treatment-associated toxicity. These strategies include cellular immunotherapies that deliver either ex vivo–expanded immune cell (such as dendritic cells [DCs]) vaccination; adoptive T-cell therapy using tumor-infiltrating lymphocytes (TILs) or chimeric antigen receptor (CAR)-engineered T cells (upper left); or in vivo–acting nanovaccines, immune checkpoint inhibitors, and cytokines (lower left). By contrast, local administration of immunostimulatory agents may result in more effective treatment at lower doses while simultaneously preventing systemic toxicity. Applying synthetic immune niches for scaffold-based adoptive cell transfer (upper right) or scaffold-based cancer vaccination (lower right) not only enables local immunomodulation, but also may overcome other limitations of current immunotherapeutic interventions that are related to cell delivery and sustained availability of immunostimulatory agents (reprinted with permission from Ref [78]; © 2018 Nature Publishing Group)

Because the anti-cancer immunotherapeutics developed to date are based on systemic delivery, they have limited therapeutic efficacy and a high risk of side effects. For example, IV infusion of an immune checkpoint inhibitor requires a high dose. By contrast, local administration can induce strong activity of anti-cancer T cells, even at low doses, while reducing treatment-associated toxicity [75–77]. Therefore, local immunomodulation provides for specific and effective cancer immunotherapy with lower associated toxicity (Fig. 5) [78]. Even when injected locally, immune cells activated at the injection site can promote systemic anti-cancer immunity. Moreover, local immunotherapy avoids the high serum antibody levels associated with systemic infusion, and thus decreases nonspecific immune cell activity, inflammatory responses, and toxicity [79, 80].

Therefore, local anti-cancer immunotherapeutics, including nanoparticles with imaging properties, have great potential. Image-guided cancer immunotherapy combined with traditional interventional oncology approaches provides a new opportunity for anti-cancer therapeutics based on nanoparticles. Image-guided local immunotherapy using nanoparticles can significantly decrease systemic toxicity by specifically delivering low-dose immunotherapeutics to solid tumors or immunological organs (e.g., lymph nodes and spleen). In this approach, imaging devices can be used to confirm whether immunotherapeutic agents are properly delivered to the target site. Cancer immunotherapeutics containing nanoparticles can also be imaged to monitor the bio-distribution of immunomodulatory agents. Image-guided local immunotherapy using nanoparticles could be applied to a wide range of cancer immunotherapeutics, including adoptive cell therapeutics, cytokines, and cancer antigens. Therefore, we believe that new anti-cancer therapeutic strategies combining nanotechnology, interventional oncology, and cancer immunotherapy will be clinically translated in the near future, enabling more effective cancer treatment than can be achieved with conventional anti-cancer therapies.

## Conclusions

This review describes the latest research aimed at improving anti-cancer immunity using nanoparticles based on biomaterials. Nanoparticles can efficiently deliver cancer antigens and adjuvants to APCs in lymph nodes, thereby aiding antigen presentation. Consequently, cancer immunotherapy using nanoparticles could yield a long-lasting vaccine effect, as well as a broader immune response than conventional immunotherapy. Recently developed mRNA-based neo-antigens are advantageous in that they have low immunogenicity but can mount an effective T-cell response when translated in the cytoplasm [81, 82]; however, such agents are susceptible to degradation by ubiquitous nucleases in the blood, and are difficult to deliver into APCs. The use of nanoparticles is a promising strategy for delivering mRNA neo-antigens to immune cells [36, 52, 83]. In addition, nanoparticles can help to restart immune surveillance by delivering immunomodulatory agents to the TME [84]. Intelligent, stimulus-responsive nanoparticles that recognize the TME could be utilized to deliver such drugs to tumor tissues with high efficiency. The therapeutic outcomes of cancer immunotherapy could also be improved by combining this approach with other modalities such as chemotherapy [85, 86], radiation therapy [87], photodynamic therapy (PDT) [88, 89], and photothermal therapy (PTT) [90, 91]. Previously, most nanoparticle-based cancer immunotherapeutics were delivered into the body via systemic administration; consequently, it was difficult to avoid systemic toxicity due to high drug doses. Hence, local cancer immunotherapy combined with traditional intentional oncology approaches holds promise for effective cancer immunotherapy because it can simultaneously decrease systemic toxicity and increase therapeutic efficacy by specifically delivering immunotherapeutics to the solid tumor or the immune system. Also, image-guided approaches could improve the success rate of cancer treatment by pre-screening patients who are highly responsive to cancer immunotherapy [92]. As biomedical technology progresses, and our understanding of the mechanisms underlying cancer immunity advances, we will develop artificial nanostructures capable of mimicking various kinds of immune cells or antibodies through nanotechnology. Recently, artificial APCs (aAPCs) have been developed as promising technologies for cancer immunotherapy [93, 94]. aAPCs synthesized using microparticles or nanoparticles can function as natural APCs to activate the adaptive immune system against cancer.

As mentioned above, recent cancer immunotherapy has involved into a convergence of multiple biomedical fields. Therefore, to develop a new concept of cancer immunotherapy it is necessary for immunologists to engage in multidisciplinary collaborations with engineers and scientists. Detailed study and understanding of the interactions between biomaterials and the immune system are needed for the design of nanomaterials suitable for anti-cancer immunotherapy. Application of biomaterial-based nanoparticles to cancer immunotherapy has the potential to improve therapeutic efficacy and diminish undesirable side effects, and thus represents an important strategy and a new direction for cancer immunotherapy. In conclusion, the development of nanoparticle-based cancer vaccines is expected to become a standard therapy capable of extending lifespan and improving quality of life in cancer patients.
